# Occupational risk factors for shoulder chronic tendinous pathology in the Spanish automotive manufacturing sector: a case-control study

**DOI:** 10.1186/s12891-020-03801-5

**Published:** 2020-12-07

**Authors:** Beatriz Rodriguez Diez-Caballero, Joaquín Alfonso-Beltrán, Iker J. Bautista, Carlos Barrios

**Affiliations:** 1 Doctorate School, Valencia Catholic University San Vicente Martir, Valencia, Spain; 2 Institute for Research on Musculoskeletal Disorders, School of Medicine and Health Sciences, Valencia Catholic University, Valencia, Spain

**Keywords:** Work-related diseases, Exposure risk, Shoulder pain, Rotator cuff injuries, Overuse, Automotive assembly industry

## Abstract

**Background:**

Musculoskeletal Diseases (MSDs) are among the most prevalent health problems encountered in the workforce in Europe. Multiple risk factors contribute to their onset. In the present study, different individual risk factors for chronic tendinous pathology affecting the shoulder were analysed in a sample of workers from the automotive manufacturing sector.

**Methods:**

An observational retrospective study was conducted with 73 cases of officially recognised and compensated occupational diseases and 94 aleatory cases of healthy workers from the same car assembly company. The experimental group comprised individuals with tendinous chronic pathology of the rotator cuff. Multiple variables that identified the risks present in the job were assessed along with participants clinical evaluation. Furthermore, two standardised guidelines for risk factors assessment were also used: the Spanish National Institute of Social Security (INSS) and the American Occupational Information Network (O*Net). Both descriptive statistical analysis and Odds ratios calculations considering the occupational disease as a dependent variable were performed.

**Results:**

The use of hand tools, exposure to mechanical pressure in the upper limbs and awkward postures were the most prevalent risk factors. Pressure on the palm of the hand and the hand tool impacting the hand were also important risk factors. Some psychosocial factors such as lack of autonomy and mental workload were also associated shoulder tendinous diseases. The association of age, load handling, and awkward postures were the core risk factors responsible for most of the tendinous chronic injuries of the shoulder in this sample of car assembly workers.

**Conclusions:**

Both ergonomic and psychosocial factors were present and increased the risk of developing occupational chronic tendinopathies at the shoulder in this sample of workers. Aging, load handling, and awkward postures showed the strongest predictive values. Greater knowledge of how risk factors interact would facilitate the design of better preventive workplace strategies.

## Introduction

Musculoskeletal disorders (MSDs) constitute a broad and complex occupational health problem in Europe, affecting a considerable portion of the working population in different sectors and occupations every year [1]. MSDs cover a wide range of conditions, from minor complaints and pains to more serious injuries requiring medical treatment and sick leave [2]. In some cases, the chronicity characteristics of MSD may even lead to permanent disabilities that prevent active work. Most work-related MSDs develop over time [1].

There is usually no single cause of MSDs; rather, various factors often work in combination, with multiple risk factors contributing to the onset of these conditions. The European Agency for Safety and Health at Work (EU-OSHA) classifies risk factors as physical, organisational, psychosocial and individual aspects [2]. Physical causes and organisational risk factors include handling loads, especially when bending and twisting, repetitive or forceful movements, awkward and static postures, vibrations, poor lighting or cold working environments, fast-paced work and prolonged sitting or standing in the same position.

The latest report issued by the European Agency for Safety and Health at Work (EU-OSHA) revealed a growing incidence of MSDs distressing millions of workers and an increasing impact on costs for employers [1]. Despite all the preventive efforts made by institutions, companies and different stakeholders working in the occupational healthcare field, the incidence of occupational diseases remains almost unchanged [3]. Data from the European Survey on Working Conditions (ESWC) reveal that the most widely-reported health problems are MSDs in the form of backache (reported by 43%) and muscular pains in the neck or upper limbs (42%) [3]. Reported health problems vary according to occupation. Skilled agricultural workers report the highest incidence of backache (57%) and muscular pains in the arms and legs. Craftspeople and workers in related trades, as well as plant and machine operators and assemblers, also report significant complaints, with more than 40% of workers in these occupations suffering from MSDs.

In Spain, and according to the Health Authorities, MSDs represent over 80% of all occupational diseases [4]. Workers in the manufacturing industry report the highest incidence rate (87.7%). The most frequent occupational diseases are overuse MSDs related to physical risk factors, mainly affecting the shoulder and the elbow. Lesion affecting the shoulder specifically included all rotator cuff tendinopathies as the most frequent diagnosed pathologies [5]. To our knowledge, there are limited data on the literature concerning the occurrence of shoulder chronic tendinopathies specifically affecting workers of the assembling automotive sector in Spain. The Spanish automotive industry is the second bigger in the world, and in Europe this sector represents around 5,6% of the total employment, this means around 12.1 million workers [5].

Until few years ago, biomechanical factors have been the most relevant in inducing work-related specific shoulder disorders. A systematic review of the available evidence in the literature at 2010 showed that highly repetitive work, forceful exertion in work, awkward postures and high psychosocial job demand are associated with the occurrence of subacromial impingement pathology [6]. No car assembly workers were included in that review. Recent research grants more and more importance to some psychosocial factors that can be directly involved as much as biomechanical consideration [7]. The most recent meta-analysis exploring which work-related risk factors are associated with specific soft tissue shoulder disorders includes a total of 16, 300 patients with specific soft tissue shoulder disorders from a population of 2, 413, 722 workers from Denmark, Finland, France, Germany and Poland. This study revealed moderate evidence for associations between shoulder disorders and arm-hand elevation, and shoulder load. Low to very low evidence was found for hand force exertion, hand-arm vibration, and psychosocial job demands. Among the 17 studies analyzed, there was only one addressing shoulder tendinopathies in workers from the US automotive sector [8].

The objective of the current study was to analyse the impact of different individual and occupational risk factors on the occurrence of shoulder chronic tendinous injuries affecting workers of large car factory. An additional the objective of the study was to create a predictive model of combined risk factors for shoulder tendinous disorders in the automotive assembly sector. This type of occupational diseases is the most common among machine operators and assembler workers according to the International Standard Classification of Occupations (ISCO). Literature addressing specific risk factor for shoulder diseases among workers of the automotive industry is relatively scarce. We hypothesise that age and exposure to the risk factors described will increase the risk of injuries, along with time employed at one’s current company. Knowledge of these risk factors and how they interact in the onset of shoulder tendinous pathologies may help to develop better preventive strategies.

## Methods

### Sample

The study comprised a group of 73 cases of shoulder occupational chronic injuries officially recognised by the regulatory health authorities between 2009 and 2014. All injured participants work in the largest car factory of Spain (Ford Valencia Body & Assembly, Almusafes, Valencia, Spain) with more than 9700 workers at the end of 2014, the last year of study. In that year, Almusafes factory was the largest European Ford plant assembling the most variety of models for many countries with a total production of more than 400,000 vehicles per year. According to the Spanish National classification of occupational diseases, all shoulder disorders are included into the same 2D0101 diagnosis code as “tendinous chronic pathology of the rotator cuff (subacromial impingement syndrome, calcifying and chronic tendinitis and rotator cuff tears)” [7]. Only these cases grouped in a single diagnosis code and showing similar clinical symptoms were included in the study. All these pathologies are also included in the code M25.811 of the International Classification of Diseases (ICD-10). Shoulder complaints could be ensured that were due to work -related tendinous chronic pathology. The reason is that all these conditions were not only specifically assessed ruling out other origins, but also recognised and compensated by the Spanish Government after a very careful follow-up procedure. A group of 94 aleatory healthy workers of the same car assembly factory constituted the control group. Healthy workers performed the same jobs under similar conditions and during the same period than the injured participants.

### Data extraction

Worker profiles and different clinical variables were retrieved from the participants records of the Safety and Health services of the car factory. Injured workers were assessed but the same Health services team composed by three different work medicine specialists that followed consensual standardized methods. These clinical records specified the characteristics of their different work activities. Variables such as sex, age, time employed at the company, time in current job, previous history of sickness, physical exercise and national occupation code (CNO) [7] were also recorded. Risk factors related to the type of job, such as manual handling of loads, repetitive movements, awkward postures (hands and arms above shoulder level), exposure to vibrations, mechanical pressure from using handheld tools, and use of personal protective equipment were regularly analysed by the same team of techniques of the Safety services of the factory as one of the their most important responsibilities within the company.

Intensity of the exposure to risk factors, and data on awkward postures, repetitive movements, physical workload, biomechanical load (repetitive movements and awkward postures), mental load and autonomy were registered following the assessment guidelines of the Spanish National Institute of Social Security [INSS] [8]. Evaluation of risk factors was also assessed by specific descriptors proposed by the American Occupational Information Network [O*Net] [9]. Among other variables, manual handling of loads, mechanical pressure from using handheld tools, use of personal protective equipment, and mental workload were registered using O*Net recommndations. Information regarding the presence or absence of each factor was converted into dichotomised variables to enable comparisons and statistical analyses.

Using the standardised measurements of occupational risk factors information provided by the Spanish INSS Guide and the O*Net network, in combination with data provided by the Safety and Health services, an objective assessment method for risk factors could be designed. In other words, it was a means of minimising some potential bias from participants regarding job conditions.

### Statistical analysis

First of all, a descriptive study of all the variables was carried out to determine the impact and extension of exposure to the different risk factors. The means and standard deviations of continuous variables were calculated, as well as the absolute and relative frequencies of categorical ones. Differences in means were calculated using one-way analysis of variance (ANOVA) tests. Categorical variables were compared using Chi-squared tests. To quantify the strength of the association between the different risk factor and the occurrence of shoulder chronic tendinous injuries, odds ratios (OR) with 95% confidence intervals were obtained considering the occupational disease as a dependent variable. Due to the large number of variables measured in the present study, a factorial analysis of principal components was done to classify risk factors according to different dimensions. Varimax rotation was implemented using a two factors approximation. Finally, regression scores of each dimension was saved. A multiple logistic regression model was used to analyse the relevance of the risk factors in combination (i.e., age, punctuations of factor one and factor two). Model adjustment was evaluated using deviance and chi^2^ test and Hosmer & Lemeshow (R^2^_L_) statistic. All analyses were performed using the SPSS 21 statistical package (IBM, Corp, Armonk, NY). For all comparisons, a two-tailed *p*-value lower than 0.05 was considered to indicate statistical significance.

## Results

The majority of those participating in the study were male (90.4% of the total participants in the study; 93% in the cases group and 88% in the control group); the mean age is 47. Five years among the cases and 38.4 years in the control group. The most frequent CNO was 7323 (adjuster and operator of machinery and tools), accounting for 50.9% of all participants, and the second most common code was 7401 (motor vehicle mechanic and fitter), accounting for 21%; in relation with this variable there are no significative differences between groups.

As for time employed at the company, there are some differences between groups, almost 80% of cases worked more than 10 years in the company but only 27% of the participants in the control group worked so long in the company; a similar result was obtained when focusing on the time spent doing the same job.

In relation to risk factors, awkward postures were the most frequent when performing work activities. In 98.6% of cases, awkward postures meant keeping one’s arms above shoulder level, and almost 99% of cases were related to repetitive forced flexion-extension movements of the shoulders. These percentages are similar to the results obtained in the control group. Furthermore, 99% of participants with a diagnose of chronic tendinous pathology of the shoulder were exposed to mechanical pressure, and 77.2% engaged in manual handling of loads.

When using standardised sources of information, the INSS shows as one of the main risk factors involved, the manual handling of loads. Physical workload was present in 65.8% of the cases at level 3, which means an intense activity equivalent to 9–12 METS, and biomechanical loading was found in 63.1% of cases (once again at level 3, meaning that it occurs during almost 60% of their working time); although physical and biomechanical workload are present in the 80% of participants that belong to the control group. Mental workload was frequently present in different ways, with lack of autonomy being present in 83% of cases and a high level of task complexity present in 98% of all participants in the study.

The O*Net network describes the manual handling of loads as the main risk factor involved in the different job descriptions reviewed along with the presence of awkward postures. The second most common risk factor was the use of handheld tools that was found in 79% of the work task descriptions. Similarly, to the Spanish INSS, the American network also explored mental workload, with the most important risk factor being lack of autonomy at work, present in 100% of job descriptions. A high level of precision tasks was present in 76.7% of job descriptions, being the second most frequent risk factor in this area. Percentages are similar in both groups.

The analysis of the association between risk factors and pathology revealed that time in job or at the company were the personal risk factors that most impacted the risk of developing an occupational disease. Thus, workers employed at the same company for between 13 and 60 months had a higher risk of developing an occupational disease, as did those who had been doing the same job for less than 3 months. A previous history of pathology and engagement in physical exercise outside work were not found to be risk factors (Table 1).

**Table 1:** Odds ratio for participants’ individual characteristics and occupational risk factors

Risk factor	OD (n:73)	CG (n:94)	OR	95% C.I.	*p*
Previous History (yes/no)	14/59	14/80	0.73	0.32–1,66	0.46
Physical Activity	31/42	43/51	1.14	0.61–2.11	0.67
Time at the company					
< 3 months	0/73	11/83	Not calculated	Not calculated	
3–6 months	0/73	1/93	Not calculated	Not calculated	
7–12 months	0/73	2/92	Not calculated	Not calculated	
13–60 months	5/68	35/59	8.06	2.96–21.92	0.00**
61–120 months	9/64	19/75	1.80	0.76–4.25	0.17
> 121 months	60/13	26/68	0.08	0.03–0.17	0.00**
Time in current job					
< 3 months	1/72	11/83	9.54	1.20–75.71	0.01*
3–6 months	5/68	3/91	0.44	0.10–1.94	0.27
7–12 months	3/70	3/91	0.76	0.15–3.92	0.75
13–60 months	16/57	30/64	1.67	0.82–3.37	0.15
61–120 months	16/57	17/77	0.78	0.36–1.68	0.53
> 121 months	32/41	31/63	0.63	0.33–1.18	0.15

[*OD* Occupational disease; *CG* Control group; *n* Number of cases; *OR* Odds ratio; *95% CI* 95% confidence interval; *p P* value].

*: *p* < 0.05; **: *p* < 0.01

The analysis of the work-related risk factors revealed that awkward postures and repetitive movements were not associated with the onset of the chronic tendinous pathology of the shoulder. However, the manual handling of loads at the second weight level (between 3 and 15 kg) did emerge as a risk factor, as did the use of light hand tools, which was found to significantly increase the risk of overuse injury.

The same result was observed in relation to exposure to mechanical pressure on the arm. When focusing specifically on concrete areas of the arm, mechanical pressure on the palm of the hand was found to pose the highest risk, followed by exertion of mechanical pressure on the fingers.

Impact by a hand tool on the heel of the hand was found to significantly increase risk, and exposure to vibrations increased the risk nearly twofold (see Table 2).

**Table 2:** Odds Ratio for occupational risk factors rom job cases

Risk Factor	OD (n:73)	CG (n:94)	OR	95% C.I.	*p*
Awkward Postures (yes/no)					
Shoulder					
Flex/Abd	72/1	88/6	0.20	0.02–1.73	0.10
High Position	72/1	88/6	0.20	0.02–1.73	0.10
Elbow					
Pronation/supination	64/9	93/1	13.07	1.60–105.7	0.02
Repetitive Movements (yes/no)	43/30	28/66	0.29	0.15–0.56	0.00
Manual Handling of Loads (yes/no)	35/38	94/0	3.68***	2.77–4.89	0.00**
< 3Kg	3/70	0/94	0.42***	1.66–43.14	0.00**
3–15 Kg	20/53	40/54	1.96	1.01–3.78	0.04*
> 15 Kg	9/64	54/40	9.6	4.27–21.55	0.00**
Use of Hand Tools (yes/no)	38/35	88/6	13.50	5.24–34.78	0.00**
< 1Kg	4/69	0/94	0.42	0.35–0.56***	0.02*
1–3 Kg	26/47	42/52	1.46	0.77–2.73	0.23
> 3Kg	7/66	46/48	9.03	3.75–21.73	0.00**
Mechanical Pressure (yes/no)	60/13	93/1	20.15	2.56–158.04	0.00**
Pressure on fingers	13/60	13/81	0.74	0.32–1.71	0.48
Pressure on palm of hand	1/72	93/1	6696	411,75–108,892	0.00
Pressure on hand	57/16	94/0	2.64***	2.15–3.25	0.00**
Vibrations	33/40	54/40	1.63	0.88–3.03	0.11
Impact on Heel of Hand	27/46	68/26	4.45	2.31–8.58	0.00**

[*OD* Occupational disease; *CG* Control group; *n* Number of cases; *OR* Odds ratio; *95% CI* 95% confidence interval; *p P* value]. *: *p* < 0.05; **: *p* < 0.01,*** OR calculated for case group.

According to the Spanish INSS Guide, the risk factors that most increased the risk of overuse injuries were biomechanical and physical workload, and lack of autonomy was found to be the most statistically significant psychosocial risk factor (see Table 3).

**Table 3:** Odds ratio for occupational risk according to the INSS and the O*Net network

Risk Factor	OD 2D0101 (n:73)	CG (n:94)	INSS OR	95% C.I.	*p*	O*Net OR	95% C.I.	*p*
Biomechanical Workload (yes/no)								
Level 2	24/49	18/76	2.06	1.01–4.2	0.04	–	–	–
Level 3	49/24	76/18	0.48	0.23–0.98	0.04			
Physical Workload								
Level 2	25/48	18/76	2.19	1.08–4.45	0.02			
Level 3	48/25	76/18	0.45	0.22–0.92	0.02	0.62	0.18–2.14	0.45
Manual Handling of loads								
Level 2	59/14	81/13	1.47	0.64–3.37	0.35			
Level 3	14/59	13/81	0.67	0.29–1.54	0.35	0.55	0.48–0.63	0.21
Mental Workload								
Level 2	35/38	46/48	0.96	0.52–1.77	0.89			
Level 3	38/35	48/46	1.04	0.56–1.91	0.89	0.82	0.40–1.70	0.60
Precision Tasks								
Level 2	2/71	2/92	1.29	0.17–9.42	0.79			
Level 3	70/3	92/2	0.50	0.08–3.11	0.45	0.89	0.42–1.85	0.75
Autonomy								
Level 2	12/61	28/66	0.46	0.21–0.99	0.04	–	–	–
Level 3	61/12	66/28	2.15	1.00–4.61	0,04			

[*OD* Occupational disease; *CG* Control group; *n* Number of cases; *OR* Odds ratio; *95% CI* 95% Confidence interval; *p P* value].

According to the data provided by the American Occupational Information Network (O*Net), exposure to precision tasks was the most important risk factor, followed by a heavy mental workload.

Factorial analysis of principal component revealed two main dimensions. The accumulated variance explained by these two factors corresponded to 36.60%. The first one dimension was in relation to load management (19.65%). The included variables and factor loading were manual handling of load [3–15 kg and > 15 kg] (0.61 and − 0.81, respectively), vibrations (0.31), physical activity (0.30) and load management (0.75). The second one dimension corresponded to awkward postures (16.95%). (i.e., shoulder Flex/Abd (0.52), high positions (0.54), elbow pronation/supination (0.52), use of hand tools (0.80), impact on heel of hand (0.47). Finally, multiple logistic regression model revealed a positive relationship between probability of shoulder injury with age, factor 1 (i.e., load management) and negative relationship with factor 2 (i.e., awkward postures). (Table 4).

**Table 4:** Summary of multiple logistical model

	Estimate	Standard Error	Z-value	***p***-value
**Intercept**	−5.972	1.235	−4.836	0.0001***
**Age**	0.124	0.026	4.658	0.0001***
**Factor 1 (i.e., load handling)**	1.638	0.329	4.965	0.0001***
**Factor 2 (i.e., awkward postures)**	−0.898	0.223	−4.030	0.0001***

***significant differences *p* < 0.0001

Deviance of null model was 228.86 on 166 degrees of freedom meanwhile deviance of residual model corresponded to 130.75 with a Chi^2^ value of 98.11 that correspond to a *p*-value < 0.0001. R^2^_L_ model was 0.43.

Odds ratio and confident interval at 95% of covariates variables were 1.13 [1.07, 1.20] for age, 5.14 [2.87, 10.66] for factor 1 (i.e., load management) and 0.41 [0.25, 0.61] for factor 2 (i.e., awkward postures).

## Discussion

At the beginning of our study we hypothesised that age and exposure to certain occupational risk factors would increase the risk of developing chronic tendinous pathology of the shoulder.

Using univariate analysis, our main results show that mean age is higher among participants in the cases group. Time at the company and time doing the same job increased the risk too. In relation to the occupational risk factors considered the main ones appeared to be awkward postures, manual handling of loads and the exposure to mechanical pressure exerted by tools. In the logistic regression model, age, load handling, and awkward postures were the core risk factors responsible for most of the tendinous chronic injuries of the shoulder in this sample of car assembly workers.

One of the potential bias of the study is common to most of the occupational health studies. Data were retrieved from clinical records. The examination of workers was not directly performed by the authors. However, the clinical exams of the workers were completed by three medical specialists of the same Health service following similar diagnostic criteria. In this study this bias was further addressed by the inclusion in the experimental group of workers officially diagnosed by Health authorities after a rigorous study. Another limitation could also be the evaluation of risk factors that was done by people from the injury prevention and safety service, but not by the authors. Expertise of these techniques in the field should be supposed. The same team analysed the different risks factor along the period of study.

Another possible bias is in relation with the perception of their jobs risks by individuals participating in the study is addressed by the use of the two standardized sources of information, fact that enhances the validity of our results.

In the present study, different individual factors were found to increase the risk of pathology. As in previous reports, ageing is a factor that increases the risk of overuse injuries in the upper extremities [10–12]. Upon analysing pooled results in this sample, it becomes clear that duration of employment increased the risk among those who had worked at the same company for more than 5 years. These findings are consistent with those reported by other authors, who reached similar conclusions [13–15].

Although our results may seem to be contradictory at this point, the contradiction may be explained by the fact that, one of the preventive measures used when one worker shows occupational disease symptoms or is diagnosed with an occupational disease, is to move him to another job within the company. This decision makes that workers don’t remain for a long time in their initial jobs but they can still work in the company for long time as they are no longer exposed to the occupational risks that harmed them.

In relation to work-related risk factors, and unlike that reported in previous studies, our results indicate that while repetitive movements and awkward postures did increase the risk slightly, the difference was not statistically significant [16, 17]. The use of hand tools (weighing more than 3 kg) increased the risk 9.03 times, a result that clearly differs from those obtained by other authors who found an OR of 1.4 [18]. Specifically, the largest OR was found when the hand tool put pressure on the palm, followed by when the mechanical pressure was exerted on the fingers [19, 20]. In any case, the use of hand tools deserves further study in order to determine its impact more precisely.

The manual handling of loads had an OR of 1.96 when the weight was between 3 and 15 kg, but the risk increased drastically (OR of 9.6) when the weight was more than 15 kg. The OR reported by the INSS was 0.67, but only for heavy loads (over 15 kg). Other authors found that the risk only increased twofold [21, 22].

Mental workload was analysed using only the information provided by the INSS and the O*Net network. Although both sources offer independent and standardised information, one of the study’s limitations is that it did not record mental workload from the workers’ perspective. Consequently, the impact of this factor on the onset of the chronic tendinous pathology of the shoulder could not be analysed. In the present study, the risk for shoulder tendinous injuries related to mental workload was slightly higher according to the INSS data, and lack of autonomy had an OR of 2.15. This result is fairly similar to those reported in other studies, and the results obtained using the O* Net information were similar to those found by authors such as Roquelaure [22], Haar [23, 25] and Haanan [24, 26].

While many studies have analysed exposure to the different risk factors affecting the working population, only a very few have focused on workers with an occupational pathology officially recognised by the public health system [17]. This is one of the strengths of the present study. All participants in the experimental group had been diagnosed by the public health authorities as having one of the occupational MSDs recognised by Spanish legislation. Furthermore, all participants in the study worked in the same area of activity, i.e. were automotive assembly employees, meaning that their work conditions and exposure to risk factors were fairly similar. Therefore, although the study includes a relatively limited number of participants, the similarity of the conditions under which they work increases the validity of the findings.

## Conclusion

Both ergonomic and psychosocial factors were present and increased the risk of developing occupational chronic tendinopathies at the shoulder in this sample of car assembly workers. The logistic regression model revealed that aging, load handling, and awkward postures were the core risk factors responsible for most of the tendinous chronic injuries of the shoulder. Manual handling of loads showed a 5-fold increase in the risk of shoulder tendinopathy. Exposure time to the different risk factors analysed in this study increases the risk of developing shoulder occupational diseases. Further studies are required with more subjects diagnosed with chronic tendinous pathology of the shoulder to confirm our findings. In the meantime, however, certain measures should be taken, including organisational changes at work, the use of other types of hand tools and the establishment of a time limit for their use by workers.

## Data Availability

The datasets used and/or analysed during the present study are available from the corresponding author upon reasonable request.

## Abbreviations

MSDs: Musculoskeletal disorders
INSS: National Institute of social security
EU-OSHA: European agency for safety and health
ESWC: European survey on working conditions
ISCO: International standard classification of occupations
CNO: National occupation code
